# Research outcomes informing the selection of public health interventions and strategies to implement them: A cross-sectional survey of Australian policy-maker and practitioner preferences

**DOI:** 10.1186/s12961-024-01144-4

**Published:** 2024-05-14

**Authors:** Luke Wolfenden, Alix Hall, Adrian Bauman, Andrew Milat, Rebecca Hodder, Emily Webb, Kaitlin Mooney, Serene Yoong, Rachel Sutherland, Sam McCrabb

**Affiliations:** 1 Faculty of Health and Medicine, School of Medicine and Public Health, University of Newcastle, Newcastle, NSW 2318 Australia; 2 Hunter New England Population Health, Hunter New England Local Health District, Wallsend, NSW 2287 Australia; 3 Hunter Medical Research Institute, Newcastle, NSW 2305 Australia; 4 Prevention Research Collaboration, Charles Perkins Centre, School of Public Health, Faculty of Medicine and Health, The University of Sydney, Sydney, NSW Australia; 5 The Australian Prevention Partnership Centre, Sydney, NSW Australia; 6 School of Public Health, University of Sydney, Sydney, NSW Australia; 7 Centre for Epidemiology and Evidence, NSW Ministry of Health, Sydney, Australia; 8 School of Health Sciences, Swinburne University of Technology, Melbourne, VIC 3122 Australia; 9 Global Nutrition and Preventive Health, Institute of Health Transformation, School of Health and Social Development, Deakin University, Burwood, VIC Australia

## Abstract

**Background:**

A key role of public health policy-makers and practitioners is to ensure beneficial interventions are implemented effectively enough to yield improvements in public health. The use of evidence to guide public health decision-making to achieve this is recommended. However, few studies have examined the relative value, as reported by policy-makers and practitioners, of different broad research outcomes (that is, measures of cost, acceptability, and effectiveness). To guide the conduct of research and better inform public health policy and practice, this study aimed at describing the research outcomes that Australian policy-makers and practitioners consider important for their decision-making when selecting: (a) public health interventions; (b) strategies to support their implementation; and (c) to assess the differences in research outcome preferences between policy-makers and practitioners.

**Method:**

An online value-weighting survey was conducted with Australian public health policy-makers and practitioners working in the field of non-communicable disease prevention. Participants were presented with a list of research outcomes and were asked to select up to five they considered most critical to their decision-making. They then allocated 100 points across these – allocating more points to outcomes perceived as more important. Outcome lists were derived from a review and consolidation of evaluation and outcome frameworks in the fields of public health knowledge translation and implementation. We used descriptive statistics to report relative preferences overall and for policy-makers and practitioners separately.

**Results:**

Of the 186 participants; 90 primarily identified as policy-makers and 96 as public health prevention practitioners. Overall, research outcomes of effectiveness, equity, feasibility, and sustainability were identified as the four most important outcomes when considering either interventions or strategies to implement them. Scores were similar for most outcomes between policy-makers and practitioners.

**Conclusion:**

For Australian policy-makers and practitioners working in the field of non-communicable disease prevention, outcomes related to effectiveness, equity, feasibility, and sustainability appear particularly important to their decisions about the interventions they select and the strategies they employ to implement them. The findings suggest researchers should seek to meet these information needs and prioritize the inclusion of such outcomes in their research and dissemination activities. The extent to which these outcomes are critical to informing the decision of policy-makers and practitioners working in other jurisdictions or contexts warrants further investigation.

**Supplementary Information:**

The online version contains supplementary material available at 10.1186/s12961-024-01144-4.

## Background

Research evidence has a key role in public health policy-making [1]. Consideration of research is important to maximize the potential impact of investments in health policies and services. Public health policy-makers and practitioners frequently seek out research to inform their professional decision-making [2]. However, they report that published research is not well aligned with their evidence needs [3, 4]. Public health decision-making is a complex and dynamic process where evidence is used in a variety of ways, and for different purposes [3, 5, 6]. Ensuring research meets the evidence needs of public health policy-makers and practitioners is, therefore, an important strategy to improve its use in decision-making [7–10].

“Research outcomes” are broad domains or constructs measured to evaluate the impacts of health policies, practices or interventions, such as their effectiveness or acceptability. They are distinct from “outcome measures”, which are the measures selected to assess an outcome. Outcome measures require detailed specification of measurement parameters, including the measurement techniques and instrument, and consideration of the suitability of its properties (for example, validity) given the research question. The inclusion of research outcomes considered most relevant to public health policy-makers and practitioners is one way in which researchers can support evidence-informed decision-making.

Policy-makers are primarily responsible for developing public health policy and selecting and resourcing health programs. Practitioners are primarily responsible for supporting their implementation. As such, public health policy-makers and practitioners require research to: (i) help identify “what works” to guide the selection of interventions that will be beneficial for their community, for example, those that are effective in improving health, and acceptable to the target population and/or (ii) to help identify “how to implement” effective intervention, for example, strategies that are capable of achieving implementation at a level sufficient to accrue benefit, are affordable and reach the targeted population [6, 11]. Research that includes outcomes relevant to these responsibilities facilitates evidence-informed decision-making by public health policy-makers and practitioners.

Initiatives such as the World Health Organization INTEGRATe Evidence (WHO INTEGRATE) framework [12], and the Grading of Recommendations Assessment, Development and Evaluation (GRADE) Evidence to Decision framework [13] have been designed to support the selection of public health interventions. Application of these frameworks required the collation and synthesis of a range of scientific evidence including studies employing qualitative and quantitative research designs. Collectively, the frameworks suggest public health policy-makers and practitioners should consider, alongside research outcomes reporting the effectiveness of a public health intervention, other research outcomes such as cost–effectiveness, potential harms and acceptability of an intervention to patients or community.

Several authors have also sought to guide outcomes researchers should include in implementation studies [11]. Proctor and colleagues defined a range of implementation research outcomes [distinct from service or clinical (intervention) effectiveness outcomes] – including intervention adoption, appropriateness, feasibility, fidelity, cost, penetration and sustainability [14]. This work helped standardize how the field of implementation science defined, measured and reported implementation outcomes. More recently McKay and colleagues put forward measures of implementation “determinants” and “outcomes” and proposed a “minimum set” of such outcomes to include in implementation and scale-up studies. The implementation research outcomes proposed by both Proctor and McKay and colleagues were developed primarily from the input of researchers to improving the quality and consistency of reporting in implementation science. However, the relative value of these outcomes to the decision-making of public health policy-makers, and in particular practitioners, has largely been unexplored.

While several studies have explored policy-maker and practitioner research evidence preferences, these have focused on a small number of potential outcomes [15–17]. An appraisal of the potential value, and importance of a comprehensive range of research outcomes to public health policy-maker and practitioner decision-making, therefore, is warranted. In this study, we sought to quantify the relative importance of research outcomes from the perspective of Australian public health policy-makers and practitioners working in the field of non-communicable disease prevention (hereafter referred to as “prevention” policy-makers or practitioners). Specifically, using a value-weighting methodology to elicit relative preferences, the study aimed to describe: (a) research the outcomes prevention policy-makers and practitioners regard as important to their decision-making when selecting a public health intervention to address an identified health issue, (b), research the outcomes prevention policy-makers and practitioners regard as important to their decision-making when selecting a strategy to support the implementation of a public health intervention in the community and (c) assess the differences between prevention policy-makers and practitioners regarding their research outcome preferences.

## Methods

### Design and setting

An online cross-sectional value-weighting survey was conducted with Australian public health prevention policy-makers and practitioners. This study was undertaken as one step of a broader program of work to establish a core outcome set that has been prospectively registered on the Core Outcome Measures in Effectiveness Trials (COMET database; https://www.comet-initiative.org/Studies/Details/1791).

### Participant eligibility

To be eligible, participants had to self-identify as having worked as a public health prevention policy-maker or practitioner at a government or non-government health organization within the past 5 years. While the term “policy-maker” has been used to describe legislators in US studies, in Australian research it has broadly been used to describe employees of government departments (or non-government agencies) involved in the development of public health policy [18–22]. Policy-makers are not typically involved in the direct implementation of policy or the delivery of health services. We defined a “policy-maker” as a professional who makes decisions, plans and actions that are undertaken to achieve specific public health prevention goals on behalf of a government or non-government organization [23]. Practitioners are typically employed by government or non-government organizations responsible for prevention service provision, and are directly involved in the implementation or supporting the implementation of public health policies or programs. Specifically, we defined a “practitioner” as a professional engaged in the delivery of public health prevention programs, implementing services or models of care in health and community settings (definition developed by research team). Research and evaluation are a core competency of the public health prevention workforce in Australia [24], as it is in other countries [25]. As such, participants may be engaged in research and have published research studies. Researchers, such as those employed by academic institutions only and without an explicit public health policy or practice role in a policy or practice organization, were excluded.

### Recruitment

Comprehensive methods were used to recruit individuals through several agencies. First, email invitations were distributed to Australian government health agencies at local (for example, New South Wales Local Health District Population Health units), state (for example, departments or ministries of health) and national levels, as well as to non-government organizations (for example, Cancer Council) and professional societies (for example, Public Health Association Australia). Registered practitioners with the International Union for Health Promotion and Education (IUHPE) from Australia were contacted by public domain emails or on LinkedIn (where identified) with the study invitation. Authors who had published articles of relevant topics from 2018 to 2021 within three Australian public health journals [*Australian and New Zealand Journal of Public Health* (*ANZJPH*), *Health Promotion Journal of Australia* (*HPJA*) and *Public Health Research and Practice* (*PHRP*)] were invited to participate in the study. Invitation emails included links to the information statement for participants and the online survey. The online survey was also promoted on the social media account of a partnering organization [National Centre of Implementation Science (NCOIS)] as well as on Twitter and LinkedIn. From these social media accounts individuals could self-select to participate in the online survey. Reminder emails were sent to non-responders at approximately 2 and 4 weeks following the initial email invitation.

### Data collection and measures

The online survey was kept on servers at the Hunter Medical Research Institute, New South Wales, Australia, and deployed using the REDCap software [26], a secured web-based application for building and managing online surveys and databases. The length of the survey was approximately 20–30 min in duration.

### Professional characteristics

Participants completed brief items assessing their professional role (that is, practitioner or policy-maker), the number of years’ experience as policy-makers or practitioners, their professional qualifications and the prevention risk factors (for example, smoking, nutrition, physical activity, injury, sexual health, etc.) for which they had expertise.

### Valued intervention and implementation outcomes

**Fig. 1:**
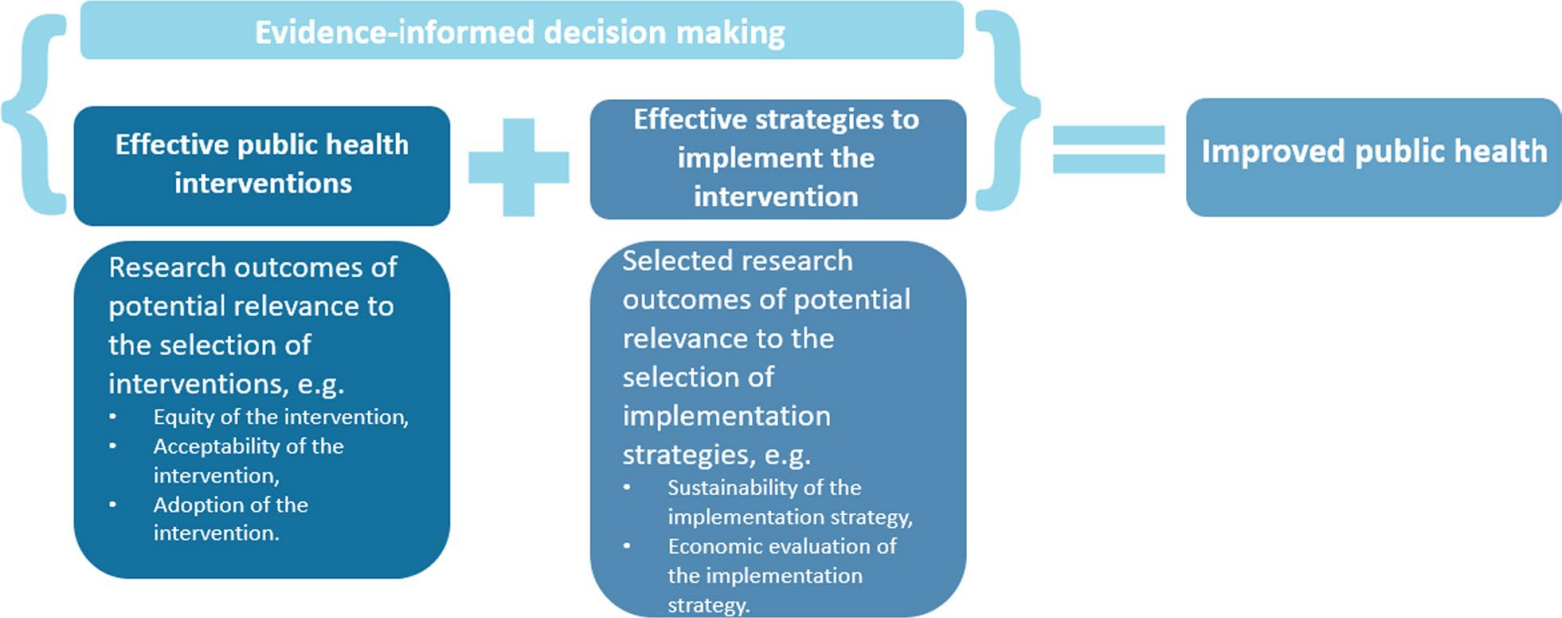
Both effective interventions and effective implementation are required to improve health outcomes

We sought to identify outcomes that may be valued by public health policy-makers and practitioners when making decisions about what policies and/or programs of interventions to implement and how implementation could best occur. We separated outcomes on this basis, consistent with recommendations of the evidence policy and practice [27], the effectiveness–implementation research typology [28, 29] and trial conduct and reporting guidelines [30]. This is illustrated in a broad study logic model (Fig. 1).

The authors undertook a review of intervention- and implementation-relevant outcome frameworks to determine program and intervention outcomes that may be of interest to policy-makers and practitioners, including the Reach, Effectiveness, Adoption, Implementation, and Maintenance (RE-AIM) Framework [31, 32], the Intervention Scalability Assessment Tool (ISAT) [18] and Proctor and colleagues’ implementation outcome definitions [14] as well as a series of publications on the topic [31, 33–43]. This was used to generate a comprehensive inventory of all possible outcomes (and outcome definitions) that may be of interest to public health policy-makers and practitioners. The outcome list was then reduced following grouping of outcomes addressing similar constructs or concepts. A panel of 16 public health policy-makers provided feedback on their perceived importance of each outcome for evidence-informed policy and practice decision-making, as well as the proposed outcome definition. This process occurred over two rounds until no further suggested improvements or clarifications were provided or requested, yielding a final list of 17 outcomes to inform the selection of public health intervention and 16 outcomes for the selection of implementation strategies (Additional file 1: Table S1). Panel participants also pre-tested the survey instrument; however, they were not invited to participate in the value-weighting study.

Participating public health policy-makers and practitioners completed the value-weighting survey. Value-weighting surveys offer advantages over other methods to identify preferences (such as ranks or mean scores on a rating scale), as they provide an opportunity to quantify the relative preference or value of different dissemination strategies from the perspective of public health policy-makers or practitioners. Specifically, they were only presented with the list of outcomes and their definition, and were asked to select up to 5 of the 17 interventions “that they considered are critical to their decision-making when selecting a public health intervention to address an identified health issue” and 16 implementation outcomes “that they consider to be critical to their decision-making when selecting a strategy to support the implementation of a public health intervention in the community” in a decision-making context. Participants were then asked to value weight, allocating 100 points across their five (or less) intervention and implementation outcomes. A higher allocation of points represented a greater level of perceived importance. In this way, participants weight the allocation of points to outcomes based on preference. No statistical weights are applied in the analysis. Participants were asked to select up to five outcomes as this restriction forced a prioritization of the outcomes among participants. The identification of a small number of critical outcomes, rather than all relevant outcomes, is also recommended to facilitate research outcome harmonization [44, 45].

### Statistical analysis

All statistical analyses and data management were undertaken in SAS version 9.3. Descriptive statistics were used to describe the study sample. Similar to other value-weighting studies, we used descriptive analyses to identify the intervention and implementation outcomes ranked from highest to lowest importance [46, 47]. Items not selected or allocated any points were assumed a score of 0, to reflect that they were not perceived as a high-priority outcome by the participant. Specifically, the mean points allocated to each of the individual outcomes were calculated and ranked in descending order. This was calculated overall for the entire participant sample, as well as separately by policy-makers and practitioners. As points were assigned in free-text fields, in instances where participants allocated more or less than 100 points across the individual items, the points they allocated were standardized to 100. Differences in the points allocated to each individual outcome by policy-maker/practitioner role were explored using Mann–Whitney *U* test. To examine any differences in the outcome preferences by participant risk factor expertise, we also examined and described outcome preferences among risk factor subgroups (with a combined sample of > 30 participants). These findings are discussed.

## Results

A total of 186 eligible participants completed the survey in part or in full.

### Professional characteristics

Of the 186 participants, 90 primarily identified as policy-makers and 96 as public health prevention practitioners (Table 1). In all, 37% of participants (47% policy-makers, 27% practitioners) had over 15 years’ experience, and approximately one third (32% policy-makers, 36% practitioners) had a PhD. The most common areas of experience were nutrition and dietetics (38% policy-maker, 53% practitioner), physical activity or sedentary behaviour (46% policy-maker, 44% practitioner), obesity (49% policy-maker, 48% practitioner) and tobacco, alcohol or other drugs (51% policy-maker, 34% practitioner).

**Table 1:** Participant characteristics

Characteristic*	Policy-maker (*n* = 90)	Practitioner (*n* = 95)	Total (*n* = 186)
Primary employer			
A government organization	52 (58%)	52 (54%)	104 (56%)
A non-government, not-for-profit organization	23 (26%)	21 (22%)	44 (24%)
A for profit organization or industry	3 (3.3%)	4 (4.2%)	7 (3.8%)
An academic institution	12 (13%)	19 (20%)	31 (17%)
Length of time working in public health			
< 5 years	10 (11%)	24 (25%)	34 (18%)
5–15 years	38 (42%)	45 (47%)	83 (45%)
15+ years	42 (47%)	26 (27%)	68 (37%)
Holds a PhD qualification	29 (32%)	34 (36%)	63 (34%)
Experience in public health topic^a^			
Nutrition and dietetics	34 (38%)	51 (53%)	85 (46%)
Physical activity of sedentary behaviour	41 (46%)	42 (44%)	83 (45%)
Overweight or obesity	44 (49%)	46 (48%)	90 (48%)
Tobacco, alcohol or other drugs	46 (51%)	33 (34%)	79 (42%)
Sexual health	22 (24%)	13 (14%)	35 (19%)
Oral health	9 (10%)	7 (7.3%)	16 (8.6%)
Injury prevention	21 (23%)	10 (10%)	31 (17%)
Violence prevention	9 (10%)	4 (4.2%)	13 (7.0%)
Mental health	33 (37%)	20 (21%)	53 (28%)
Infectious diseases	20 (22%)	13 (14%)	33 (18%)

^*^Cell totals may not add up to total sample size due to missing values

^a^Cells do not add up to 100% as participants could select more than one area of experience

### Valued outcomes

#### Intervention outcomes

**Fig. 2:**
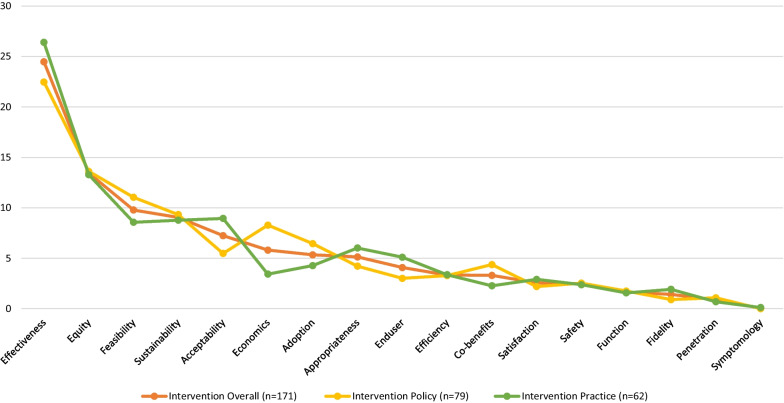
Line graph representing mean points allocated for the 17 intervention outcomes overall and by role

A total of 169 participants (83 policy-makers and 86 practitioners, with 7 and 10 missing, respectively) responded to the value-weighting questions for the 17 listed intervention outcomes. Table 2 (Fig. 2) reports the mean and standard deviation of points allocated by policy-makers and practitioners for each outcome, ranked in descending order to represent the most to least important. For policy-makers and practitioners combined, the effectiveness of an intervention, and its impact on equity, were clearly identified by participants as the leading two outcomes, with a mean allocation of 24.47 [standard deviation (SD) = 17.43] and 13.44(SD = 12.80), respectively. The mean scores for outcomes of feasibility (9.78) and sustainability (9.04) that ranked third and fourth, respectively, were similar; then scores dropped noticeably to 7.24 for acceptability and 5.81 for economic outcomes.

**Table 2:** Mean points allocated for each of the 17 intervention outcomes overall and by role

Outcome	Policy-makers (*n* = 83)		Practitioners (*n* = 86)		All (*n* = 169)	
	Mean (SD)	Rank	Mean (SD)	Rank	Mean (SD)	Rank
Effectiveness of the intervention	22.47 (16.74)	1	26.40 (17.95)	1	24.47 (17.43)	1
Equity of the intervention	13.61 (13.67)	2	13.28 (11.99)	2	13.44 (12.80)	2
Feasibility of the intervention	11.04 (11.74)	3	8.57 (9.56)	5	9.78 (10.73)	3
Sustainability of the intervention	9.33 (10.59)	4	8.77 (9.93)	4	9.04 (10.23)	4
Acceptability of the intervention	5.48 (9.62)	7	8.95 (9.11)	3	7.24 (9.49)	5*
Economic assessments of the intervention	8.28 (10.73)	5	3.43 (6.56)	9	5.81 (9.16)	6*
Adoption of the intervention	6.45 (9.16)	6	4.27 (8.15)	8	5.34 (8.70)	7
Appropriateness of the intervention	4.22 (8.82)	9	6.02 (9.43)	6	5.13 (9.15)	8
Intervention end-user-centredness	3.01 (6.98)	11	5.10 (9.55)	7	4.08 (8.43)	9
Efficiency of the intervention	3.28 (8.21)	10	3.37 (7.99)	10	3.33 (8.08)	10
Co-benefits of the intervention	4.37 (7.78)	8	2.27 (6.49)	13	3.30 (7.21)	11*
Satisfaction with an intervention	2.20 (5.95)	13	2.91 (8.42)	11	2.56 (7.30)	12
Intervention adverse effects and safety	2.53 (8.28)	12	2.38 (6.07)	12	2.46 (7.22)	13
Individual- (that is, patient-) reported and function-based outcomes of the intervention	1.75 (5.71)	14	1.57 (5.16)	15	1.66 (5.42)	14
Intervention fidelity	0.90 (3.76)	16	1.92 (6.05)	14	1.42 (5.06)	15
Intervention penetration	1.08 (4.94)	15	0.69 (3.39)	16	0.88 (4.22)	16
Symptomatology	0.00 (0.00)	17	0.12 (1.08)	17	0.06 (0.77)	17

*Statistically significant difference in the mean allocation of points by policy-makers and practitioners based on Mann–Whitney *U* test

For most outcomes, average scores were similar for policy-makers and practitioners. However, practitioner scores for the outcome of acceptability (mean = 8.95, SD = 9.11), which ranked third most important for practitioners was significantly different than for policy-makers (mean = 5.48, SD = 9.62), where it was ranked seventh (*p* = 0.005). Economics/cost outcomes were ranked fifth by policy-makers (mean = 8.28, SD = 10.63), which significantly differed from practitioners (mean = 3.43, SD = 6.56), where it was ranked ninth (*p* = 0.002). For co-benefits, ranked eighth by policy-makers (mean = 4.37, SD = 7.78), scores were significantly different than for practitioners (mean = 2.27, SD = 6.49), where it was ranked thirteenth (*p* = 0.0215). Rankings for the top five outcomes were identical for those with expertise in nutrition and dietetics, physical activity or sedentary behaviour, obesity and tobacco, alcohol or other drugs (Additional file 1: Table S2).

#### Implementation outcomes

**Fig. 3:**
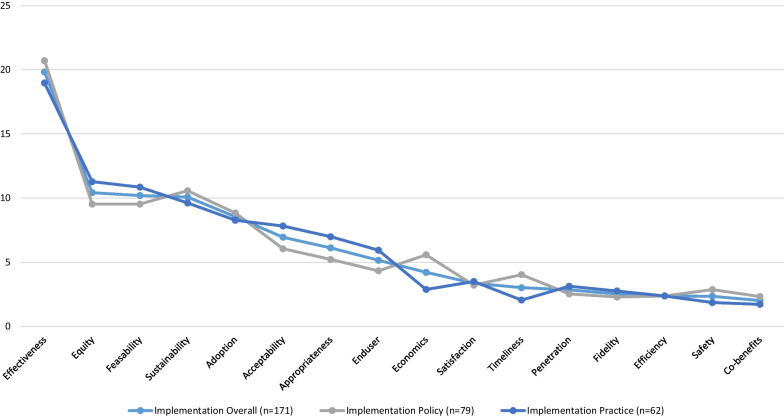
Line graph representing mean points allocated for the 16 implementation outcomes overall and by role

A total of 153 participants (75 policy-makers and 78 practitioners, with 15 and 18 missing, respectively) responded to the value-weighting questions for the 16 listed implementation outcomes (Table 3, Fig. 3). The effectiveness of an implementation strategy was clearly identified by participants as the most important intervention outcome, with a mean allocation of 19.82 (SD = 16.85) overall. The mean scores for the next three ranked outcomes namely equity (mean = 10.42, SD = 12.7), feasibility (mean = 10.2, SD = 12.91) and sustainability (mean = 10.08, SD = 10.58) were similar, and thereafter, scores noticeably dropped for measures of adoption (mean = 8.55, SD = 10.90), the fifth-ranked outcome.

**Table 3:** Mean points allocated for all 16 implementation outcomes overall and by role

Outcome: definition	Policy-maker (*n* = 75)		Practitioner (*n* = 78)		Overall (*n* = 153)	
	Mean (SD)	Rank	Mean (SD)	Rank	Mean (SD)	Rank
Effectiveness of implementation strategy on the intervention implementation	20.71 (16.63)	1	18.97 (17.12)	1	19.82 (16.85)	1
Equity of implementation strategy	9.53 (13.36)	3	11.28 (12.05)	2	10.42 (12.70)	2
Feasibility of implementation strategy	9.53 (11.44)	4	10.85 (14.23)	3	10.20 (12.91)	3
Sustainability of the implementation strategy	10.57 (11.15)	2	9.62 (10.06)	4	10.08 (10.58)	4
Adoption of the implementation strategy	8.84 (11.14)	5	8.27 (10.72)	5	8.55 (10.90)	5
Acceptability of the implementation strategy**	6.05 (9.18)	6	7.82 (11.67)	6	6.95 (10.52)	6
Appropriateness of the implementation strategy	5.22 (8.72)	8	6.99 (9.95)	7	6.12 (9.38)	7
End-user-centredness of the implementation strategy	4.33 (9.31)	9	5.94 (10.55)	8	5.15 (9.96)	8
Economic evaluation of the implementation strategy	5.58 (9.25)	7	2.88 (6.67)	11	4.21 (8.12)	9*
Satisfaction with the implementation strategy	3.22 (6.87)	11	3.50 (8.31)	9	3.36 (7.61)	10
Timeliness of the implementation strategy	4.03 (7.72)	10	2.05 (5.78)	14	3.02 (6.85)	11
Penetration of the implemented practice	2.53 (7.94)	13	3.13 (7.13)	10	2.84 (7.52)	12
Fidelity of implementation strategy	2.29 (6.05)	16	2.76 (7.88)	12	2.53 (7.02)	13
Efficiency of the implementation strategy	2.37 (5.83)	14	2.37 (6.87)	13	2.37 (6.36)	14
Safety and adverse effects of the implementation strategy	2.87 (7.08)	12	1.86 (6.35)	15	2.35 (6.71)	15
Co-benefits of the implementation	2.33 (7.13)	15	1.72 (6.17)	16	2.02 (6.65)	16

**The label was provided but the incorrect definition was used when surveying participants. Acceptability of the implementation strategy was incorrectly defined as “A measure of the uptake or reach of an implementation strategy” in the survey

*Statistically significant difference in the mean allocation of points by policy-makers and practitioners based on Mann–Whitney *U* test

For most implementation outcomes (Fig. 3) policy-makers and practitioners scores were similar. However, economics outcomes were ranked seventh for policy-makers with a mean = 5.58 (SD = 9.25), compared with practitioners who had a ranking of eleventh for this outcome (mean = 2.88, SD = 6.67). The difference in the points allocated were statistically significant between the two groups (*p* = 0.0439). Timeliness was ranked tenth most important for policy-makers, with a mean allocation of 4.03 (SD = 7.72), compared with practitioners who had a ranking of fourteenth for this outcome and a mean allocation of 2.05 (SD = 5.78). The difference in mean scores between policy-makers and practitioners on this outcome was not significant. Rankings and scores were similar for those with expertise in nutrition and dietetics, physical activity or sedentary behaviour, obesity and tobacco, alcohol or other drugs (Additional file 1: Table S3).

## Discussion

Broadly, this study sought to better understand the information valued by public health policy-makers and practitioners to support their decisions regarding what and how interventions should be implemented in the community. The most valued research outcomes were the same regardless of whether policy-makers or practitioners were selecting interventions or implementation strategies. Namely outcomes regarding the effectiveness of interventions and implementation strategies. Following this, outcomes about equity, feasibility and sustainability also appeared to represent priorities. The study also found broad convergence among the most valued research outcomes, between policy-makers and practitioners, and across participants with expertise across different non-communicable disease (NCD) risk factors (for example, nutrition, obesity and tobacco). Such findings underscore the importance of research reporting these outcomes to support the translation of public health research into policy and practice.

For outcomes about decisions regarding intervention selection, the findings are broadly consistent with factors recommended by evidence-to-decision frameworks. For example, the top six ranked outcomes (effectiveness, equity, feasibility, sustainability, acceptability and economic), are also represented in both the WHO INTEGRATE framework [12] and the GRADE Evidence to Decision framework [13]. However, research outcomes about harms (adverse effects), which are included in both the WHO INTEGRATE and GRADE frameworks were ranked 13th by participants in this study. Such a finding was surprising given that potential benefits and harms of an intervention must be considered to appraise its net impact on patient or public health. Health professionals, however, do not have accurate expectations of the harms and benefits of therapeutic interventions. This appears particularly to be the case for public health professionals who acknowledge the potential for unintended consequences of policies [48] but consider these risks to be minimal [49]. The findings, therefore, may reflect the tendency of health professionals to overestimate the benefits of therapeutic interventions, and to a larger extent, underestimate harms [50, 51]. In doing so, participants may have elevated their reported value of outcomes regarding the beneficial effectiveness of an intervention and discounted their value of outcomes reporting potential harms. Further research is warranted to substantiate this hypothesis, or explore whether other factors such as participant comprehension or misinterpretation of the outcome description may explain the finding. Nonetheless, the inclusion of measures of adverse effects (or harms) as trial outcomes is prudent to support evidence-informed public health decision-making, as is the use of strategies to facilitate risk communication to ensure the likelihood of such outcomes is understood by policy-makers and practitioners [52–54].

To our knowledge, this is the first study to examine the research evidence needs of public health policy-makers and practitioners when deciding on what strategies may be used to support policy or program implementation. Most of the eight implementation outcomes recommended by Proctor and colleagues [14] were ranked within the top eight by participants of this study. However, equity outcomes, ranked second by these participants, were not an outcome included in the list of outcomes defined by Proctor and colleagues. The findings may reflect public health values, which, as a discipline, has equity at its core [55]. It may also reflect the increasing attention to issues of health equity in implementation science [56].

Further, one of the eight Proctor outcomes, penetration – defined by Proctor and colleagues as the integration or saturation of an intervention within a service setting and its subsystems – was not ranked highly. Successful penetration implies a level of organization institutionalization of an intervention, which once achieved may continue to provide ongoing benefit to patients or populations. It may also suggest the capacity within the organization to expand implementation or adopt new interventions. Penetration outcomes, therefore, have been suggested to be particularly important to model and understand the potential impact of investment of scarce health resources in the implementation of public health policies and interventions [57].

At face value, such findings may suggest, at least from the perspective of public health policy-makers and practitioners, that penetration outcomes may not be particularly valued in terms of decision-making. However, it may also reflect a lack of familiarity with this term among public health policy-makers and practitioners, where related outcomes such as “reach” are more commonly used in the literature [14, 58]. Alternatively it may be due to the conceptual similarity of this and other outcomes such as adoption, maintenance or sustainability. In other studies, for example, penetration has been operationalized to include the product of “reach”, “adoption” and “organizational maintenance” [58]. A lack of clear conceptual distinction may have led some participants to allocate points to related outcomes such as “adoption” rather than “penetration”.

The use of concept mapping techniques, consolidation of definitions of existing outcomes, and articulation of specific measures aligned to these outcomes may reduce some of these conceptual challenges. Indeed, best practice processes to develop core outcome sets for clinical trials suggest processes of engagement with end-users [45], stakeholders and researchers to articulate both broad outcomes and specific measures of these to support a shared understanding of important outcomes (and measures) to be included in such research. For example, there are many measures and economic methods to derive related to a broad outcome of “cost” (for example, absolute costs, cost–effectiveness, cost–benefit, cost–utility, and budget impact analysis) [59]. However, public health policy-makers’ preference or perceived value of these different measures to their decision-making will likely vary. While work in the field to map or align specific measures to broad outcomes is ongoing [57, 58, 60], extending this to empirically investigate end-user preferences for measures would be an important contribution to the field.

Broadly speaking, there was little variation in the outcomes valued between policy-makers and practitioners. However, economic evaluations were ranked as more important by policy-makers. The findings may reflect differences in the roles of Australian public health policy-makers and practitioners. That is, government policy-makers are often responsible for setting and financing the provision of public health programs, whereas health practitioners are responsible for directly supporting or undertaking their delivery. Economic considerations, therefore, may have greater primacy among policy-makers, who may be more likely to incur program costs [19]. Further research to explore and better understand these areas of divergence is warranted.

The study intended to provide information about outcomes that were generally of most use in public health policy and practice decision-making. However, such decisions are often highly contextual, and preferences may vary depending on the policy-maker or practitioner, the health issue to be addressed, the target population or broader decision-making circumstances [2, 61]. As such, the extent to which the findings reported in this study generalize to other contexts, such as those working in different fields of public health, on different health issues or from countries or jurisdictions outside Australia is unknown. Future research examining the outcome preferences of public health policy-makers and practitioners in different contexts, therefore, is warranted.

The contextual nature of evidence needs of policy-makers and practitioners may explain, in part, the variability in outcome preferences. In many cases, for example, the mean of the outcome preference was less than its standard deviation. The interpretation of the study findings should consider this variability. That is, there is little distinguishing the mean preference ranks of many outcomes. However, the study findings at the extremes are unambiguous, suggesting clear preferences for the highest over the lowest ranking outcomes that did not differ markedly across policy-makers, practitioners or those with expertise in addressing different non-communicable disease risks such as nutrition, physical activity or tobacco or alcohol use.

Several study limitations are worth considering when interpreting the research findings. The initial inventory of outcomes was compiled from outcome frameworks, many of which were generic health or medical research outcomes that are uncommon in public health prevention research. There was considerable overlap in the outcomes included across frameworks, though how these were defined at times varied. Variability in outcome terminology has previously been identified as a problem for the field [62]. Despite being provided definitions for each, some participants may have responded to survey items based on their pre-existing understanding of these terms. Furthermore, following completion of the study, a programming error was identified whereby the definition of “Acceptability of the implementation strategy” was incorrectly assigned as “A measure of the uptake or reach of an implementation strategy”. The extent to which this may have influenced participant preferences is unclear, so sensitivity analysis was conducted by removing all participants who selected acceptability as a measure of interest. We conducted two analyses, one where the people who chose acceptability were removed but their other rankings remained and another where all their data were deleted. Results indicated that the top five outcomes did not differ after conducting the analysis, with only sustainability moving from fourth to second place in the second sensitivity analysis (Additional file 1: Tables S4 and S5).

## Conclusion

The pathway from research production to research in health policy or practice is complex. While a range of effective public health policies and interventions exist across a range of community settings [63–66], their implementation at a level capable of achieving population-level risk reductions remains elusive [67–70]. Nonetheless, undertaking research with end-use in mind, including reporting of outcomes valued by decision-makers, will likely facilitate the knowledge translation process [7]. In this study we found that outcomes related to effectiveness, equity, feasibility and sustainability appear important to decisions policy-makers and practitioners make about the interventions they select and the strategies they employ to implement public health prevention initiatives. Researchers interested in supporting evidence-informed decision-making should seek to provide for these information needs and prioritize such outcomes in dissemination activities to policy-makers and practitioners.

## Contribution to the literature

- It is essential to the research needs of policy-makers and practitioners to determine core outcomes to facilitate research use and knowledge translation.
- Here we quantify the relative values of a variety of research outcomes commonly used in health research.
- Findings suggest the primary outcomes of interest to public health prevention policy-makers and practitioners when making decisions about the selection of interventions and strategies to implement them are related to effectiveness, equity, feasibility and sustainability and that these do not differ markedly between public health prevention policy-makers and practitioners.

### Supplementary Information

**Additional file 1: Table S1.** Mean point allocations for each of the 17 intervention outcomes overall and by area of expertise (where field of expertise n ≥ 30).** Table S2.** Mean point allocations for each of the 16 implementation outcomes overall and by area of expertise (where field of expertise n ≥ 30).** Table S3.** Mean points for implementation outcomes overall and by area of expertise (field of expertise n ≥ 30).** Table S4.** Sensitivity analysis, participants who selected ‘acceptability’ removed from the analysis, their other rankings remained.** Table S5.** Sensitivity analysis, participants who selected ‘acceptability’ whole data set removed from the analysis.

## Data Availability

The datasets used and/or analysed during the current study are available from the corresponding author on reasonable request.

## Abbreviations

*ANZJPH*: *Australian and New Zealand Journal of Public Health*
*HPJA*: *Health Promotion Journal of Australia*
ISAT: Intervention scalability assessment tool
NCD: Non-communicable disease
NCOIS: National Centre of Implementation Science
*PHRP*: *Public Health Research and Practice*
WHO: World Health Organization
