# Supplementary material for: Research outcomes informing the selection of public health interventions and strategies to implement them: A cross-sectional survey of Australian policy-maker and practitioner preferences
Source: Health Res Policy Syst. 2024 May 14;22:58. doi: 10.1186/s12961-024-01144-4 (PMC11095011; doi:10.1186/s12961-024-01144-4)
Supplement: Supplementary file 1 — Additional file 1: Table S1. Mean point allocations for each of the 17 intervention outcomes overall and by area of expertise (where field of expertise n ≥ 30). Table S2. Mean point allocations for each of the 16 implementation outcomes overall and by area of expertise (where field of expertise n ≥ 30). Table S3. Mean points for implementation outcomes overall and by area of expertise (field of expertise n ≥ 30). Table S4. Sensitivity analysis, participants who selected ‘acceptability’ removed from the analysis, their other rankings remained. Table S5. Sensitivity analysis, participants who selected ‘acceptability’ whole data set removed from the analysis. [file 12961_2024_1144_MOESM1_ESM.docx]

| **Outcome** | **Definition** |
| --- | --- |
| **Intervention outcomes** | |
| The effectiveness or impact of the intervention on the targeted health behaviour or condition | A measure of how well an intervention performs in addressing the targeted health behaviour or condition (includes effectiveness and efficacy research). |
| Equity of the intervention | The extent to which an intervention is fair, inclusive and does not exacerbate health inequities. |
| Feasibility of the intervention | The extent to which an intervention can be successfully used or carried out within a given agency, setting or timeframe. |
| Sustainability of the intervention | Continued use of intervention components, following intervention completion, for the continued achievement of desirable outcomes. |
| Acceptability of the intervention | A measure of the extent to which people delivering or receiving an intervention consider it to be acceptable. |
| Economic assessments of the intervention | Comparative analyses of the intervention and alternative courses of action in terms of both their costs and benefits. |
| Adoption of the intervention: | A measure of the uptake or reach of an intervention. |
| Appropriateness of the intervention | The perceived fit of an intervention to end-users and the broader system where it is to be delivered. |
| Intervention end-user-centredness | A measure of the extent to which an intervention is respectful of and responsive to individual or organisational preferences, needs, and values. This may include individual or patient reports of care received or intervention. |
| Efficiency of the intervention | The maximum output obtained for a given intervention from a given set of resources |
| Co-benefits of the intervention | The impact of the intervention on non-health outcomes. This may include the environmental, climate, and social impacts of an intervention. |
| Satisfaction with an intervention | A measure of how well an intervention fulfils desires and needs of end-users. |
| Intervention adverse effects and safety | Measure of the extent to which an intervention does not expose individuals to danger, or risk of harm. |
| Individual (i.e. patient) reported and function based outcomes of the intervention | A measure of a patient’s functional status such as their ability to perform daily activities required to meet basic needs, fulfil usual roles, and maintain health and well-being? |
| Intervention fidelity | The degree to which an intervention was implemented as it was prescribed or as intended by the program developers. |
| Intervention penetration | A measure of the extent to which an intervention is integrated within a service setting and its subsystems. |
| Symptomatology | The set of symptoms characteristic of a medical condition or exhibited by an individual or patient. |
| **Implementation outcomes** | |
| Effectiveness of implementation strategy on the intervention implementation | A measure of how well an implementation strategy performs in addressing the targeted health behaviour or condition |
| Equity of implementation strategy | An implementation strategy that is fair and impartial within a given context and does not exacerbate health inequities. |
| Feasibility of implementation strategy | The extent to which an implementation strategy can be successfully used or carried out within a given agency, setting or timeframe. |
| Sustainability of the implementation strategy | The extent to which a newly implemented implementation strategy is maintained within a service setting’s ongoing, stable operations. |
| Adoption of the implementation strategy | A measure of how well a policy/program/implementation strategy has been taken up by implementers and/or ‘end users’. |
| Acceptability of the implementation strategy | A measure of the extent to which people delivering or receiving an intervention consider it to be acceptable.** |
| Appropriateness of the implementation strategy | The perceived fit of an implementation strategy with end-users to the broader system where it is to be delivered. |
| End-user-centredness of the implementation strategy | Providing an implementation strategy that is respectful of and responsive to individual or organisational preferences, needs, and values and ensuring that patient values guide all decisions regarding the program/policy. |
| Economic evaluation of the implementation strategy | The comparative analysis of alternative courses of implementation in terms of both their costs and benefits. |
| Satisfaction with the implementation strategy | A measure of how well the implementation strategy fulfils desires and needs of end-users. |
| Timeliness of the implementation strategy | A measure of how an implementation strategy is delivered within a window where it is responding to a priority issue and is resourced, welcomed by users and addressing a specific identified need. |
| Penetration of the implemented practice | The integration of an implementation strategy within a service setting and its subsystems. |
| Fidelity of implementation strategy | The degree to which an implementation strategy was implemented as it was prescribed in the original protocol or as it was intended by the program developers. |
| Efficiency of the implementation strategy | The maximum output obtained for an implementation strategy from a given set of resources. |
| Safety and adverse effects of the implementation strategy | An implementation strategy that protects individuals or implementing organisations from danger, risk of harm. |
| Co-benefits of the implementation | The impact of the implementation on non-health outcomes (e.g. the environmental, climate, and social impacts of an intervention). |

** The label was provided but the incorrect definition was used when surveying participants. Acceptability of the implementation strategy was incorrectly defined as “A measure of the uptake or reach of an implementation strategy” in the survey.

**Supplementary file**

**Table 1.** Mean point allocations for each of the 17 intervention outcomes overall and by area of expertise (where field of expertise n≥30).

| **Response option** | **Nutrition and dietetics (n=79)** | | **Physical activity or Sedentary behaviour (n=76)** | | **Overweight or obesity (n=84)** | | **Tobacco, Alcohol or Other drugs (n=73)** | | **Sexual health (n=31)** | | **Mental health (n=46)** | | **Infectious disease (n=31)** | | **Other (n=30)** | | **Overall (n=169)** | |
| --- | --- | --- | --- | --- | --- | --- | --- | --- | --- | --- | --- | --- | --- | --- | --- | --- | --- | --- |
|  | **Mean (SD)** | **Rank** | **Mean (SD)** | **Rank** | **Mean (SD)** | **Rank** | **Mean (SD)** | **Rank** | **Mean (SD)** | **Rank** | **Mean (SD)** | **Rank** | **Mean (SD)** | **Rank** | **Mean (SD)** | **Rank** | **Mean (SD)** | **Rank** |
| The effectiveness or impact of the intervention on the targeted health behaviour or condition: A measure of how well an intervention performs in addressing the targeted health behaviour or condition (includes effectiveness and efficacy research). | 24.01 (16.45) | 1 | 23.41 (17.59) | 1 | 22.88 (16.44) | 1 | 24.21 (16.71) | 1 | 23.71 (16.73) | 1 | 22.59 (17.44) | 1 | 21.77 (17.49) | 1 | 19.50 (18.59) | 1 | 24.47 (17.43) | 1 |
| Equity of the intervention: The extent to which an intervention is fair, inclusive and does not exacerbate health inequities. | 15.78 (12.39) | 2 | 14.39 (12.87) | 2 | 14.15 (11.93) | 2 | 14.57 (13.88) | 2 | 15.48 (14.28) | 2 | 15.30 (15.24) | 2 | 12.26 (12.70) | 2 | 14.00 (12.83) | 2 | 13.44 (12.80) | 2 |
| Feasibility of the intervention: The extent to which an intervention can be successfully used or carried out within a given agency, setting or timeframe. | 9.88 (9.70) | 3 | 9.82 (10.35) | 3 | 9.81 (10.06) | 3 | 10.95 (11.09) | 3 | 8.42 (9.97) | 5 | 7.93 (9.22) | 4 | 10.97 (10.28) | 3 | 12.33 (14.61) | 3 | 9.78 (10.73) | 3 |
| Sustainability of the intervention: Continued use of intervention components, following intervention completion, for the continued achievement of desirable outcomes. | 9.42 (10.24) | 4 | 9.07 (10.34) | 4 | 9.80 (9.85) | 4 | 9.84 (10.69) | 4 | 12.26 (11.02) | 3 | 9.31 (11.56) | 3 | 8.87 (12.63) | 4 | 9.33 (10.40) | 4 | 9.04 (10.23) | 4 |
| Acceptability of the intervention: A measure of the extent to which people delivering or receiving an intervention consider it to be acceptable. | 7.93 (10.34) | 5 | 7.52 (9.11) | 5 | 8.32 (10.71) | 5 | 7.11 (9.01) | 5 | 6.84 (10.24) | 6 | 6.90 (9.67) | 5 | 6.45 (10.50) | 6 | 7.33 (9.26) | 5 | 7.24 (9.49) | 5 |
| Economic assessments of the intervention: Comparative analyses of the intervention and alternative courses of action in terms of both their costs and benefits. | 5.53 (8.51) | 6 | 7.20 (9.54) | 6 | 5.68 (8.51) | 6 | 5.16 (8.68) | 7 | 5.55 (8.64) | 7 | 4.78 (8.43) | 9 | 6.45 (9.15) | 7 | 5.33 (8.70) | 8 | 5.81 (9.16) | 6 |
| Adoption of the intervention: A measure of the uptake or reach of an intervention. | 5.38 (8.58) | 7 | 5.00 (8.56) | 8 | 5.26 (8.79) | 8 | 5.99 (9.50) | 6 | 3.39 (6.76) | 9 | 6.52 (9.48) | 6 | 4.84 (8.32) | 9 | 4.83 (8.35) | 9 | 5.34 (8.70) | 7 |
| Appropriateness of the intervention: The perceived fit of an intervention to end-users and the broader system where it is to be delivered. | 5.32 (9.11) | 8 | 5.26 (8.98) | 7 | 5.62 (9.24) | 7 | 5.10 (9.71) | 8 | 8.55 (12.33) | 4 | 5.65 (8.54) | 8 | 6.94 (9.46) | 5 | 6.17 (12.23) | 6 | 5.13 (9.15) | 8 |
| Intervention end-user-centredness: A measure of the extent to which an intervention is respectful of and responsive to individual or organisational preferences, needs, and values. This may include individual or patient reports of care received or intervention. | 5.05 (9.39) | 9 | 4.13 (8.14) | 9 | 4.51 (8.49) | 9 | 4.23 (7.94) | 9 | 3.39 (7.46) | 10 | 3.56 (7.35) | 10 | 5.97 (10.52) | 8 | 4.00 (8.14) | 10 | 4.08 (8.43) | 9 |
| Efficiency of the intervention: The maximum output obtained for a given intervention from a given set of resources. | 2.18 (6.79) | 11 | 2.99 (7.76) | 11 | 2.94 (7.82) | 11 | 2.63 (6.84) | 12 | 2.26 (6.69) | 12 | 0.43 (2.06) | 16 | 1.94 (4.77) | 13 | 3.50 (7.09) | 12 | 3.33 (8.08) | 10 |
| Co-benefits of the intervention: The impact of the intervention on non-health outcomes. This may include the environmental, climate, and social impacts of an intervention. | 2.53 (6.09) | 10 | 3.16 (6.87) | 10 | 3.01 (6.84) | 10 | 3.19 (6.98) | 10 | 3.55 (7.09) | 8 | 5.72 (9.71) | 7 | 3.06 (6.79) | 12 | 6.00 (10.37) | 7 | 3.30 (7.21) | 11 |
| Satisfaction with an intervention: A measure of how well an intervention fulfils desires and needs of end-users. | 2.13 (5.63) | 12 | 2.61 (6.70) | 12 | 2.36 (6.46) | 12 | 0.86 (3.13) | 15 | 0.81 (3.19) | 14 | 3.37 (7.53) | 11 | 3.39 (10.52) | 11 | 2.50 (10.57) | 13 | 2.56 (7.30) | 12 |
| Intervention adverse effects and safety: Measure of the extent to which an intervention does not expose individuals to danger, or risk of harm. | 1.84 (7.03) | 13 | 2.43 (7.68) | 13 | 2.20 (7.17) | 13 | 3.15 (8.72) | 11 | 2.74 (6.81) | 11 | 2.93 (7.19) | 12 | 4.68 (8.75) | 10 | 0.33 (1.83) | 15 | 2.46 (7.22) | 13 |
| Individual (i.e. patient) reported and function based outcomes of the intervention: A measure of a patient’s functional status such as their ability to perform daily activities required to meet basic needs, fulfil usual roles, and maintain health and well-being? | 0.76 (3.50) | 16 | 1.58 (5.67) | 14 | 1.43 (5.41) | 14 | 1.37 (4.81) | 14 | 2.26 (6.17) | 13 | 2.83 (6.80) | 13 | 1.61 (5.23) | 14 | 0.83 (3.24) | 14 | 1.66 (5.42) | 14 |
| Intervention fidelity: The degree to which an intervention was implemented as it was prescribed or as intended by the program developers. | 0.95 (4.32) | 15 | 0.72 (3.80) | 15 | 1.01 (4.58) | 15 | 1.44 (4.68) | 13 | 0.32 (1.80) | 16 | 1.30 (5.42) | 14 | 0.00 (0.00) | 16 | 3.83 (8.27) | 11 | 1.42 (5.06) | 15 |
| Intervention penetration: A measure of the extent to which an intervention is integrated within a service setting and its subsystems. | 1.32 (5.26) | 14 | 0.72 (3.62) | 16 | 1.01 (4.71) | 16 | 0.21 (1.76) | 16 | 0.48 (2.69) | 15 | 0.87 (4.12) | 15 | 0.81 (3.67) | 15 | 0.17 (0.91) | 16 | 0.88( 4.22) | 16 |
| Symptomatology: The set of symptoms characteristic of a medical condition or exhibited by an individual or patient. | 0.00 (0.00) | 17 | 0.00 (0.00) | 17 | 0.00 (0.00) | 17 | 0.00 (0.00) | 17 | 0.00 (0.00) | 17 | 0.00 (0.00) | 17 | 0.00 (0.00) | 17 | 0.00 (0.00) | 17 | 0.06 (0.77) | 17 |

**Table 2.** Mean point allocations for each of the 16 implementation outcomes overall and by area of expertise (where field of expertise n≥30).

| **Response option** | **Nutrition and dietetics (n=73)** | | **Physical activity or Sedentary behaviour (n=70)** | | **Overweight or obesity (n=77)** | | **Tobacco, Alcohol or Other drugs (n=62)** | | **Mental health (n=43)** | | **Overall (n=153)** | |
| --- | --- | --- | --- | --- | --- | --- | --- | --- | --- | --- | --- | --- |
|  | **Mean (SD)** | **Rank** | **Mean (SD)** | **Rank** | **Mean (SD)** | **Rank** | **Mean (SD)** | **Rank** | **Mean (SD)** | **Rank** | **Mean (SD)** | **Rank** |
| Effectiveness of implementation strategy on the intervention implementation: A measure of how well an implementation strategy performs in a real world setting where variables cannot be controlled. | 18.56 (15.58) | 1 | 18.71 (15.90) | 1 | 18.57 (15.37) | 1 | 20.05 (17.09) | 1 | 23.56 (15.44) | 1 | 19.82 (16.85) | 1 |
| Equity of implementation strategy: An implementation strategy that is fair and impartial within a given context and does not exacerbate health inequities. | 10.82 (12.33) | 4 | 9.79 (11.96) | 3 | 9.55 (12.09) | 4 | 11.77 (13.88) | 2 | 11.16 (14.22) | 2 | 10.42 (12.70) | 2 |
| Feasibility of implementation strategy: The extent to which an implementation strategy can be successfully used or carried out within a given agency, setting or timeframe. | 11.86 (14.53) | 2 | 9.71 (9.85) | 4 | 10.97 (14.19) | 2 | 10.45 (11.72) | 3 | 7.62 (9.55) | 4 | 10.20 (12.91) | 3 |
| Sustainability of the implementation strategy: The extent to which a newly implemented implementation strategy is maintained within a service setting’s ongoing, stable operations. | 11.58 (10.99) | 3 | 10.07 (10.44) | 2 | 10.78 (10.88) | 3 | 10.21 (10.65) | 4 | 10.30 (10.14) | 3 | 10.08 (10.58) | 4 |
| Adoption of the implementation strategy: A measure of how well a policy/program/implementation strategy has been taken up by implementers and/or ‘end users’. | 9.27 (11.96) | 5 | 8.52 (11.06) | 5 | 8.53 (11.20) | 5 | 8.68 (9.28) | 5 | 7.55 (9.04) | 5 | 8.55 (10.90) | 5 |
| Acceptability of the implementation strategy: A measure of the extent to which people delivering or receiving an intervention consider it to be acceptable.** | 5.78 (8.95) | 7 | 6.24 (9.52) | 7 | 6.52 (9.59) | 6 | 7.28 (9.80) | 6 | 5.66 (8.86) | 7 | 6.95 (10.52) | 6 |
| Appropriateness of the implementation strategy: The perceived fit of an implementation strategy with end-users to the broader system where it is to be delivered. | 4.52 (8.17) | 8 | 6.71 (9.89) | 6 | 5.97 (9.36) | 8 | 6.05 (9.54) | 7 | 6.90 (10.12) | 6 | 6.12 (9.38) | 7 |
| End-user-centredness of the implementation strategy: Providing an implementation strategy that is respectful of and responsive to individual or organisational preferences, needs, and values and ensuring that patient values guide all decisions regarding the program/policy. | 6.44 (11.26) | 6 | 5.14 (9.44) | 8 | 6.04 (10.98) | 7 | 3.71 (8.59) | 9 | 3.95 (7.91) | 10 | 5.15 (9.96) | 8 |
| Economic evaluation of the implementation strategy: The comparative analysis of alternative courses of implementation in terms of both their costs and benefits. | 3.70 (7.99) | 10 | 4.57 (8.29) | 9 | 3.87 (8.04) | 10 | 3.96 (7.81) | 8 | 3.98 (8.17) | 9 | 4.21 (8.12) | 9 |
| Satisfaction with the implementation strategy: A measure of how well the implementation strategy fulfils desires and needs of end-users. | 4.04 (8.32) | 9 | 3.86 (8.08) | 11 | 3.96 (8.17) | 9 | 3.06 (7.21) | 10 | 5.62 (9.78) | 8 | 3.36 (7.61) | 10 |
| Timeliness of the implementation strategy: A measure of how an implementation strategy is delivered within a window where it is responding to a priority issue and is resourced, welcomed by users and addressing a specific identified need. | 3.36 (6.82) | 11 | 4.43 (8.06) | 10 | 3.66 (7.06) | 11 | 2.85 (6.95) | 12 | 2.95 (6.77) | 12 | 3.02 (6.85) | 11 |
| Penetration of the implemented practice: The integration of an implementation strategy within a service setting and its subsystems. | 3.22 (8.39) | 12 | 3.14 (8.17) | 12 | 2.60 (7.59) | 12 | 2.90 (6.87) | 11 | 2.44 (7.02) | 14 | 2.84 (7.52) | 12 |
| Fidelity of implementation strategy: The degree to which an implementation strategy was implemented as it was prescribed in the original protocol or as it was intended by the program developers. | 1.53 (4.90) | 15 | 2.52 (6.68) | 13 | 2.42 (6.46) | 14 | 2.85 (6.80) | 13 | 1.78 (4.86) | 15 | 2.53 (7.02) | 13 |
| Efficiency of the implementation strategy: The maximum output obtained for an implementation strategy from a given set of resources. | 2.40 (6.93) | 13 | 2.07 (6.84) | 16 | 2.53 (7.05) | 13 | 2.14 (5.47) | 15 | 0.58 (2.72) | 16 | 2.37 (6.36) | 14 |
| Safety and adverse effects of the implementation strategy: An implementation strategy that protects individuals or implementing organisations from danger, risk of harm. | 1.37 (4.81) | 16 | 2.29 (6.41) | 14 | 1.75 (5.11) | 16 | 2.18 (6.44) | 14 | 2.67 (6.67) | 13 | 2.35 (6.71) | 15 |
| Co-benefits of the implementation: The impact of the implementation on non-health outcomes (e.g. the environmental, climate, and social impacts of an intervention). | 1.56 (6.33) | 14 | 2.21 (7.83) | 15 | 2.27 (7.76) | 15 | 1.85 (7.85) | 16 | 3.26 (9.75) | 11 | 2.02 (6.65) | 16 |

** The label was provided but the incorrect definition was used when surveying participants. Acceptability of the implementation strategy was incorrectly defined as “A measure of the uptake or reach of an implementation strategy” in the survey.

Table 3. Mean points for implementation outcomes overall and by area of expertise (field of expertise n≥30)

| **Response option** | **Nutrition and dietetics (n=73)** | | **Physical activity or Sedentary behaviour (n=70)** | | **Overweight or obesity (n=77)** | | **Tobacco, Alcohol or Other drugs (n=62)** | | **Mental health (n=43)** | | **Overall (n=153)** | |
| --- | --- | --- | --- | --- | --- | --- | --- | --- | --- | --- | --- | --- |
|  | **Mean (SD)** | **Rank** | **Mean (SD)** | **Rank** | **Mean (SD)** | **Rank** | **Mean (SD)** | **Rank** | **Mean (SD)** | **Rank** | **Mean (SD)** | **Rank** |
| Effectiveness of implementation strategy on the intervention implementation | 18.56 (15.58) | 1 | 18.71 (15.90) | 1 | 18.57 (15.37) | 1 | 20.05 (17.09) | 1 | 23.56 (15.44) | 1 | 19.82 (16.85) | 1 |
| Equity of implementation strategy | 10.82 (12.33) | 4 | 9.79 (11.96) | 3 | 9.55 (12.09) | 4 | 11.77 (13.88) | 2 | 11.16 (14.22) | 2 | 10.42 (12.70) | 2 |
| Feasibility of implementation strategy | 11.86 (14.53) | 2 | 9.71 (9.85) | 4 | 10.97 (14.19) | 2 | 10.45 (11.72) | 3 | 7.62 (9.55) | 4 | 10.20 (12.91) | 3 |
| Sustainability of the implementation strategy | 11.58 (10.99) | 3 | 10.07 (10.44) | 2 | 10.78 (10.88) | 3 | 10.21 (10.65) | 4 | 10.30 (10.14) | 3 | 10.08 (10.58) | 4 |
| Adoption of the implementation strategy | 9.27 (11.96) | 5 | 8.52 (11.06) | 5 | 8.53 (11.20) | 5 | 8.68 (9.28) | 5 | 7.55 (9.04) | 5 | 8.55 (10.90) | 5 |
| Acceptability of the implementation strategy** | 5.78 (8.95) | 7 | 6.24 (9.52) | 7 | 6.52 (9.59) | 6 | 7.28 (9.80) | 6 | 5.66 (8.86) | 7 | 6.95 (10.52) | 6 |
| Appropriateness of the implementation strategy | 4.52 (8.17) | 8 | 6.71 (9.89) | 6 | 5.97 (9.36) | 8 | 6.05 (9.54) | 7 | 6.90 (10.12) | 6 | 6.12 (9.38) | 7 |
| End-user-centredness of the implementation strategy | 6.44 (11.26) | 6 | 5.14 (9.44) | 8 | 6.04 (10.98) | 7 | 3.71 (8.59) | 9 | 3.95 (7.91) | 10 | 5.15 (9.96) | 8 |
| Economic evaluation of the implementation strategy | 3.70 (7.99) | 10 | 4.57 (8.29) | 9 | 3.87 (8.04) | 10 | 3.96 (7.81) | 8 | 3.98 (8.17) | 9 | 4.21 (8.12) | 9 |
| Satisfaction with the implementation strategy | 4.04 (8.32) | 9 | 3.86 (8.08) | 11 | 3.96 (8.17) | 9 | 3.06 (7.21) | 10 | 5.62 (9.78) | 8 | 3.36 (7.61) | 10 |
| Timeliness of the implementation strategy | 3.36 (6.82) | 11 | 4.43 (8.06) | 10 | 3.66 (7.06) | 11 | 2.85 (6.95) | 12 | 2.95 (6.77) | 12 | 3.02 (6.85) | 11 |
| Penetration of the implemented practice | 3.22 (8.39) | 12 | 3.14 (8.17) | 12 | 2.60 (7.59) | 12 | 2.90 (6.87) | 11 | 2.44 (7.02) | 14 | 2.84 (7.52) | 12 |
| Fidelity of implementation strategy | 1.53 (4.90) | 15 | 2.52 (6.68) | 13 | 2.42 (6.46) | 14 | 2.85 (6.80) | 13 | 1.78 (4.86) | 15 | 2.53 (7.02) | 13 |
| Efficiency of the implementation strategy | 2.40 (6.93) | 13 | 2.07 (6.84) | 16 | 2.53 (7.05) | 13 | 2.14 (5.47) | 15 | 0.58 (2.72) | 16 | 2.37 (6.36) | 14 |
| Safety and adverse effects of the implementation strategy | 1.37 (4.81) | 16 | 2.29 (6.41) | 14 | 1.75 (5.11) | 16 | 2.18 (6.44) | 14 | 2.67 (6.67) | 13 | 2.35 (6.71) | 15 |
| Co-benefits of the implementation | 1.56 (6.33) | 14 | 2.21 (7.83) | 15 | 2.27 (7.76) | 15 | 1.85 (7.85) | 16 | 3.26 (9.75) | 11 | 2.02 (6.65) | 16 |

** The label was provided but the incorrect definition was used when surveying participants. Acceptability of the implementation strategy was incorrectly defined as “A measure of the uptake or reach of an implementation strategy” in the survey.

Of 153 participants who selected the implementation strategies and had valid data to be included in the value weighting analysis, 58 selected acceptability as one of their top 5 outcomes. In Table 4, their acceptability data is removed but their other rankings retained.

To note, only 22 out of the 153 (14%) selected both acceptability and adoption as part of their top 5.

**Table 4.** Sensitivity analysis, participants who selected ‘acceptability’ removed from the analysis, their other rankings remained.

| **Outcome** | **Policy maker (n=75)** | | **Practitioner (n=78)** | | **Overall (n=153)** | |
| --- | --- | --- | --- | --- | --- | --- |
|  | **mean (SD)** | **Rank** | **mean (SD)** | **Rank** | **mean (SD)** | **Rank** |
| Effectiveness of implementation strategy A measure of how well an implementation strategy performs in a real world setting. | 22.01(17.46) | 1 | 20.58(18.88) | 1 | 21.28(18.15) | 1 |
| Equity of implementation strategy An implementation strategy that is fair and impartial within a given context. | 10.34(14.46) | 3 | 11.97(12.64) | 2 | 11.17(13.54) | 2 |
| Feasibility of implementation strategy The extent to which an implementation strategy can be successfully used or carried out within a given agency, setting or timeframe. | 9.98(12.02) | 4 | 11.85(14.98) | 3 | 10.93(13.60) | 3 |
| Sustainability of the implementation strategy The extent to which a newly implemented implementation strategy is maintained within a service setting’s ongoing, stable operations. | 11.15(11.80) | 2 | 10.21(10.71) | 4 | 10.67(11.23) | 4 |
| Adoption of the implementation strategy A measure of how well a policy/program has been taken up by implementers and/or ‘end users’. | 9.26(11.67) | 5 | 8.88(11.48) | 5 | 9.07(11.54) | 5 |
| Appropriateness of the implementation strategy The perceived fit of an implementation strategy with end-users to the broader system where it is to be delivered. | 5.67(9.57) | 7 | 7.76(11.12) | 6 | 6.73(10.41) | 6 |
| End-user-centredness of the implementation strategy Providing an implementation strategy that is respectful of and responsive to individual or organisational preferences, needs, and values and ensuring that patient values guide all decisions regarding the | 4.70(10.15) | 8 | 6.41(11.45) | 7 | 5.57(10.83) | 7 |
| Economic evaluation of the implementation strategy The comparative analysis of alternative courses of implementation in terms of both their costs and benefits.* | 6.12(10.24) | 6 | 2.99(6.90) | 11 | 4.52(8.81) | 8 |
| Satisfaction with the implementation strategy A measure of how well the implementation strategy fulfils desires and needs of end-users. | 3.49(7.57) | 10 | 4.03(9.50) | 8 | 3.77(8.58) | 9 |
| Timeliness of the implementation strategy A measure of how an implementation strategy is delivered within a window where it is responding to a priority issue and is resourced, welcomed by users and addressing a specific identified need. | 4.17(8.09) | 9 | 2.17(6.10) | 14 | 3.15(7.19) | 10 |
| Penetration of the implemented practice The integration of an implementation strategy within a service setting and its subsystems. | 2.53(7.94) | 13 | 3.16(7.18) | 9 | 2.85(7.55) | 11 |
| Fidelity of implementation strategy The degree to which an implementation strategy was implemented as it was prescribed in the original protocol or as it was intended by the program developers. | 2.58(6.97) | 12 | 3.03(8.61) | 10 | 2.81(7.83) | 12 |
| Safety of the implementation strategy An implementation strategy that protects individuals or implementing organisations from danger, risk of harm. | 3.12(7.86) | 11 | 2.25(7.37) | 13 | 2.68(7.60) | 13 |
| Efficiency of the implementation strategy The maximum output obtained for an implementation strategy from a given set of resources. | 2.44(5.96) | 14 | 2.87(8.34) | 12 | 2.66(7.26) | 14 |
| Co-benefits of the implementation. The impact of the implementation on non-health outcomes (e.g. the environmental, climate, and social impacts of an intervention). | 2.43(7.36) | 15 | 1.83(6.63) | 15 | 2.13(6.98) | 15 |

*Statistically significant difference between roles

Of 153 participants who selected the implementation strategies and had valid data to be included in the value weighting analysis, 58 selected acceptability as one of their top 5 outcomes. In Table 5, all of their data is removed.

**Table 5.** Sensitivity analysis, participants who selected ‘acceptability’ whole data set removed from the analysis.

| **Outcome** | **Policy maker (n=50)** | | **Practitioner (n=45)** | | **Overall (n=95)** | |
| --- | --- | --- | --- | --- | --- | --- |
|  | **mean (SD)** | **Rank** | **mean (SD)** | **Rank** | **mean (SD)** | **Rank** |
| Effectiveness of implementation strategy A measure of how well an implementation strategy performs in a real world setting. | 22.06(17.61) | 1 | 16.67(14.14) | 1 | 19.51(16.21) | 1 |
| Equity of implementation strategy An implementation strategy that is fair and impartial within a given context. | 12.56(11.40) | 2 | 12.33(10.20) | 3 | 12.45(10.79) | 2 |
| Feasibility of implementation strategy The extent to which an implementation strategy can be successfully used or carried out within a given agency, setting or timeframe. | 9.60(13.99) | 4 | 13.56(12.78) | 2 | 11.47(13.50) | 3 |
| Sustainability of the implementation strategy The extent to which a newly implemented implementation strategy is maintained within a service setting’s ongoing, stable operations. | 10.45(10.88) | 3 | 11.24(17.11) | 4 | 10.83(14.11) | 4 |
| Adoption of the implementation strategy A measure of how well a policy/program has been taken up by implementers and/or ‘end users’. | 9.53(10.68) | 5 | 9.00(11.61) | 5 | 9.28(11.07) | 5 |
| Appropriateness of the implementation strategy The perceived fit of an implementation strategy with end-users to the broader system where it is to be delivered. | 4.60(8.26) | 8 | 5.78(9.59) | 7 | 5.16(8.89) | 6 |
| End-user-centredness of the implementation strategy Providing an implementation strategy that is respectful of and responsive to individual or organisational preferences, needs, and values and ensuring that patient values guide all decisions regarding the | 4.20(9.28) | 9 | 5.84(9.81) | 6 | 4.98(9.52) | 7 |
| Economic evaluation of the implementation strategy The comparative analysis of alternative courses of implementation in terms of both their costs and benefits. | 4.81(8.47) | 7 | 4.11(7.85) | 10 | 4.48(8.15) | 8 |
| Satisfaction with the implementation strategy A measure of how well the implementation strategy fulfils desires and needs of end-users. | 3.80(9.51) | 10 | 4.87(8.64) | 8 | 4.31(9.07) | 9 |
| Timeliness of the implementation strategy A measure of how an implementation strategy is delivered within a window where it is responding to a priority issue and is resourced, welcomed by users and addressing a specific identified need. | 4.84(8.08) | 6 | 2.89(6.78) | 11 | 3.92(7.52) | 10 |
| Penetration of the implemented practice The integration of an implementation strategy within a service setting and its subsystems. | 3.20(6.76) | 11 | 4.40(9.38) | 9 | 3.77(8.09) | 11 |
| Fidelity of implementation strategy The degree to which an implementation strategy was implemented as it was prescribed in the original protocol or as it was intended by the program developers. | 2.95(6.38) | 12 | 2.33(6.09) | 14 | 2.66(6.22) | 12 |
| Safety of the implementation strategy An implementation strategy that protects individuals or implementing organisations from danger, risk of harm. | 2.60(6.33) | 14 | 2.44(7.81) | 13 | 2.53(7.03) | 13 |
| Efficiency of the implementation strategy The maximum output obtained for an implementation strategy from a given set of resources. | 2.90(8.15) | 13 | 1.87(5.45) | 15 | 2.41(6.99) | 14 |
| Co-benefits of the implementation. The impact of the implementation on non-health outcomes (e.g. the environmental, climate, and social impacts of an intervention). | 1.90(5.43) | 15 | 2.67(8.43) | 12 | 2.26(6.99) | 15 |
